# Moderate Hyponatremia Is Associated with Increased Risk of Mortality: Evidence from a Meta-Analysis

**DOI:** 10.1371/journal.pone.0080451

**Published:** 2013-12-18

**Authors:** Giovanni Corona, Corinna Giuliani, Gabriele Parenti, Dario Norello, Joseph G. Verbalis, Gianni Forti, Mario Maggi, Alessandro Peri

**Affiliations:** 1 Endocrinology Unit, Maggiore-Bellaria Hospital, Bologna, Italy; 2 Endocrine Unit, “Center for Research, Transfer and High Education on Chronic, Inflammatory, Degenerative and Neoplastic Disorders for the Development of Novel Therapies” (DENOThe), Dept. of Experimental and Clinical Biomedical Sciences, University of Florence, Careggi Hospital, Florence, Italy; 3 Andrology Unit, “Center for Research, Transfer and High Education on Chronic, Inflammatory, Degenerative and Neoplastic Disorders for the Development of Novel Therapies” (DENOThe), Dept. of Experimental and Clinical Biomedical Sciences, University of Florence, Careggi Hospital, Florence, Italy; 4 Division of Endocrinology and Metabolism, Georgetown University, Washington, DC, United States of America; Bambino Gesù Children Hospital, Italy

## Abstract

**Background:**

Hyponatremia is the most common electrolyte disorder in clinical practice, and evidence to date indicates that severe hyponatremia is associated with increased morbidity and mortality. The aim of our study was to perform a meta-analysis that included the published studies that compared mortality rates in subjects with or without hyponatremia of any degree.

**Methods and Findings:**

An extensive Medline, Embase and Cochrane search was performed to retrieve the studies published up to October 1^st^ 2012, using the following words: “hyponatremia” and “mortality”. Eighty-one studies satisfied inclusion criteria encompassing a total of 850222 patients, of whom 17.4% were hyponatremic. The identification of relevant abstracts, the selection of studies and the subsequent data extraction were performed independently by two of the authors, and conflicts resolved by a third investigator. Across all 81 studies, hyponatremia was significantly associated with an increased risk of overall mortality (RR = 2.60[2.31–2.93]). Hyponatremia was also associated with an increased risk of mortality in patients with myocardial infarction (RR = 2.83[2.23–3.58]), heart failure (RR = 2.47[2.09–2.92]), cirrhosis (RR = 3.34[1.91–5.83]), pulmonary infections (RR = 2.49[1.44–4.30]), mixed diseases (RR = 2.59[1.97–3.40]), and in hospitalized patients (RR = 2.48[2.09–2.95]). A mean difference of serum [Na^+^] of 4.8 mmol/L was found in subjects who died compared to survivors (130.1±5.6 *vs* 134.9±5.1 mmol/L). A meta-regression analysis showed that the hyponatremia-related risk of overall mortality was inversely correlated with serum [Na^+^]. This association was confirmed in a multiple regression model after adjusting for age, gender, and diabetes mellitus as an associated morbidity.

**Conclusions:**

This meta-analysis shows for the first time that even a moderate serum [Na^+^] decrease is associated with an increased risk of mortality in commonly observed clinical conditions across large numbers of patients.

## Introduction

Hyponatremia, defined as a serum sodium concentration ([Na^+^]) <136 mmol/L, is the most common electrolyte disorder encountered in clinical practice [1]. The most common cause of hypotonic or dilutional hyponatremia is the syndrome of inappropriate antidiuresis (SIAD). Mild hyponatremia (serum [Na^+^] 130–135 mmol/L) has been estimated to occur in about 15–30% of hospitalized patients, whereas the prevalence of moderate to severe hyponatremia (serum [Na^+^] <130) is as high as 7% among in-hospital patients [2].

Hyponatremia represents a serious health problem with significant associated morbidity and mortality. Acute severe hyponatremia is a medical emergency accompanied by severe neurological symptoms due to cerebral edema and can be lethal if not recognized and appropriately treated [3]. The correction of hyponatremia may *per se* represent a risk and a rare but potentially lethal complication, i.e. the osmotic demyelination syndrome, may be the result of an overly rapid correction [4]. In contrast, mild chronic hyponatremia has traditionally been considered as an asymptomatic or mildly symptomatic condition. However, recent reports indicated that even mild chronic hyponatremia can have long-term adverse effects, such as deficits in gait and attention [5], falls [5], bone loss and fractures [6]–[9], especially in the elderly. More recently, chronic hyponatremia has been shown to exacerbate multiple manifestation of senescence in aged rats, including senile osteoporosis, sarcopenia, cardiac fibrosis, and hypogonadism [10].

The association between hyponatremia and in-hospital mortality has been demonstrated in numerous studies. For instance, a large cohort study, which included all adult hospitalizations (n = 53236) at an academic medical center between 2000–2007, demonstrated that even mild hyponatremia was associated with increased in-hospital mortality, and that the risk of death was increased by 2.3% for each 1 mmol/L decline of serum [Na^+^] [11].

Hyponatremia has been generally associated with an increased mortality in different conditions such as pneumonia [12], heart failure [13], acute myocardial infarction [14], cirrhosis [15], cancer [14], in the elderly [16], and in intensive care patients [17]. However, whether hyponatremia is an independent risk factor for death or is simply associated with an underlying severe condition that is the cause of death remains to be elucidated [4], [18]. Furthermore, there is the possibility that hyponatremia indirectly contributes to mortality by causing organ dysfunction, such as for example bone loss and fractures which are associated with significant mortality in the elderly. Recently, a meta-analysis that included 22 observational studies and randomized controlled trials published to the end of 2008, that was limited to patients with heart failure, indicated that hyponatremia is a powerful predictor of mortality in these patients regardless of ejection fraction [19]. However, no meta-analysis on the relationship between hyponatremia and mortality has addressed other pathological conditions to date.

The aim of this study was to perform a meta-analysis, which included the studies that compared the mortality rate in subjects with or without hyponatremia, in order to verify whether hyponatremia represents a risk factor for mortality, independently of other confounding factors.

## Methods

A meta-analysis was performed including studies comparing mortality rate in subjects with or without hyponatremia. An extensive Medline, Embase, and Cochrane search was performed including the following words: hyponatremia and mortality. The search up to October 1^st^ 2012 was restricted to English-language articles and studies of human participants. The identification of relevant abstracts, the selection of studies based on the criteria described above, and the subsequent data extraction were performed independently by two of the authors (G.P., C.G.), and conflicts resolved by a third investigator (G.C). Full-text articles and meeting abstracts were included. The quality of studies was assessed using the Cochrane criteria [20].

### Statistical analysis

Heterogeneity was assessed using the I^2^ statistics for overall mortality rate. Considering that heterogeneity could not be excluded (I^2^ = 92.8%), relative risk of mortality between subjects with or without hyponatremia, was calculated using both a random and fixed effect model. For a more conservative approach, results of random effect models were presented. A meta-regression analysis was performed to test the effect of serum [Na^+^] threshold selected in the different studies on overall mortality rate levels. In addition, a linear regression analysis model, weighing each study for the number of subjects enrolled, was performed to verify the independent effect of hyponatremia on mortality after the adjustment for age, gender and diabetes mellitus as an associated morbidity. It was not possible to include other co-morbidities because there were not enough data to be collected and analyzed from the selected literature. Finally, sensitivity analyses was performed considering only larger studies (including ≥1000 subjects) or those reporting the prevalence of diabetes mellitus. In addition, mean baseline serum [Na^+^] in subjects who eventually died or not at follow up were meta-analyzed using a random effect model.

Relative risks (RRs) with 95% CIs were calculated using Comprehensive Meta-analysis Version 2, Biostat, (Englewood, NJ, USA). Logistic multivariate analysis was performed on SPSS (Statistical Package for the Social Sciences; Chicago, USA) for Windows 20.1.

## Results

**Figure 1:**
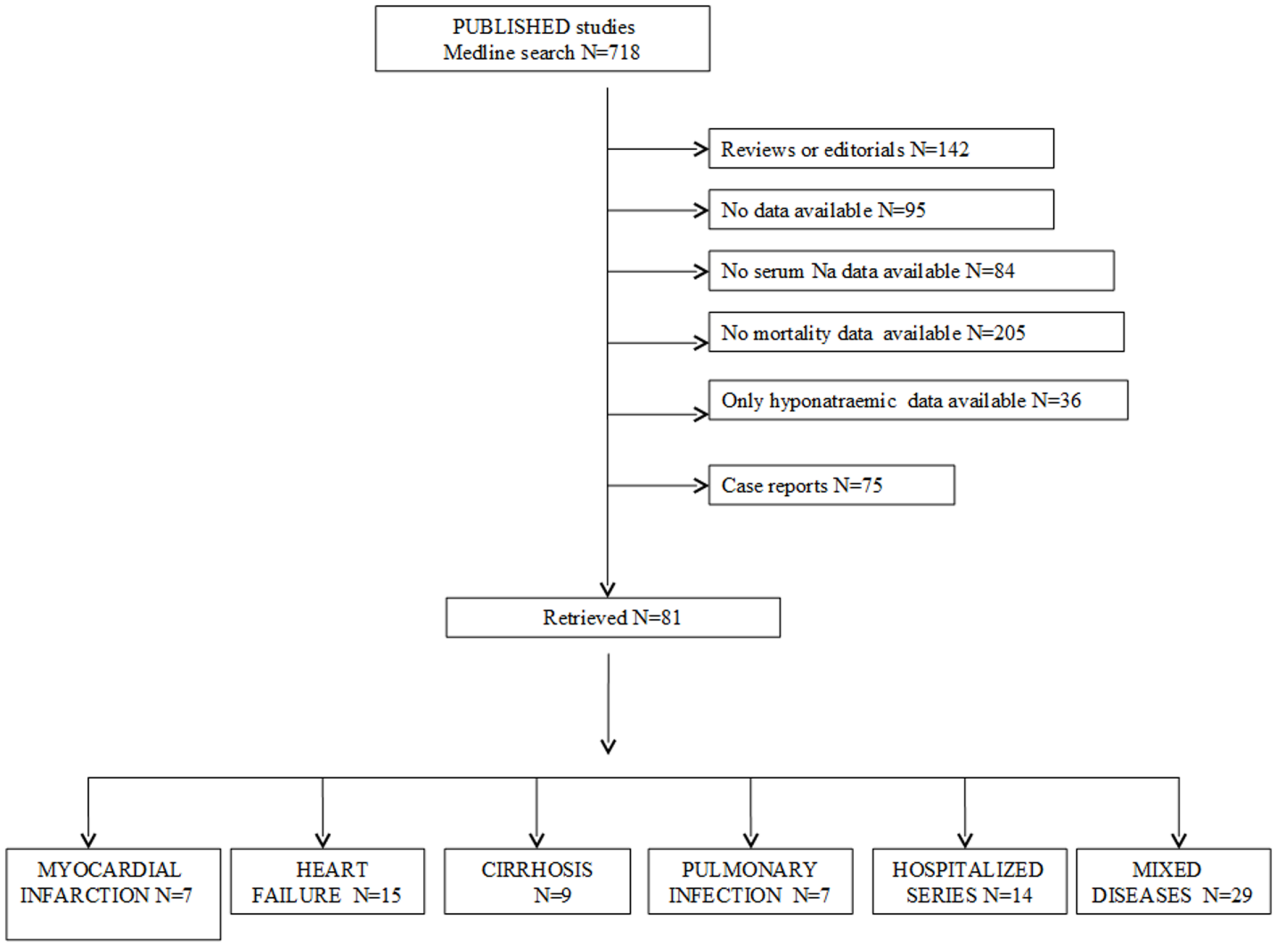
Trial flow diagram.

Out of 718 retrieved articles, 637 articles were excluded for different reasons. The flow of the meta-analysis is summarized in Figure 1, and the characteristics of the trials included in the meta-analysis are summarized in Table 1 (see references 3,11–12,16–18, 21–95). Among the 81 selected studies, 7, 13, 8, 5 studies evaluated the effect of hyponatremia on overall mortality rate in subjects with myocardial infarction, heart failure (HF), cirrhosis and pulmonary infections, respectively. In addition, another 26 studies reported data on the effect of hyponatremia on overall mortality for combined mixed diseases, which could not be grouped separately (see Table 1). Finally, 14 studies retrospectively investigated the effect of hyponatremia on overall mortality in hospitalized series of subjects. In these studies, a major diagnosis was not specified.

**Table 1:** Studies included in meta-analysis.

Source	Type of disease	Age	Male	DM	Na^+^ cut-off (mEq/L)	Patients	H	NH	Deaths H	DeathsNH	Na^+^ deaths (mEq/L)	Na^+^ survivors
		(years)	%	%		(n)	(n)	(n)	(n)	(n)	(mean±SD)	(mEq/L) (mean±SD)
Flear et al., 1979 [21]	Myocardial infarction	57.1	78.7	NA	135	235	88	147	19	10	NA	NA
Goldberg et al., 2004 [22]	Myocardial infarction	61	78	24.2	135	1047	339	708	61	44	NA	NA
Goldberg et al., 2006 [23]	Myocardial infarction	59.3	80.7	22.6	136	978	108	870	26	78	NA	NA
Klopotowski et al., 2009 [24]	Myocardial infarction	NA	72.5	8.9	135	1858	96	1762	13	67	NA	NA
Havrànek et al., 2011 [25]	Myocardial infarction	64	66	33.9	135	218	72	146	25	30	NA	NA
Tada et al., 2011 [26]	Myocardial infarction	64.4	85	41.4	136	140	29	111	0	3	NA	NA
Tang et al., 2011 [27]	Myocardial infarction	63.8	6.8	2.9	135	1620	212	1408	29	103	NA	NA
Panciroli et al., 1990 [28]	HF	67	70.2	11.8	135	161	64	97	44	39	NA	NA
Adewole et al., 1996 [29]	HF	NA	NA	NA	125	64	10	54	7	17	NA	NA
Chen et al., 2003 [30]	HF	56	63.2	NA	125	234	27	207	20	35	NA	NA
Villacorta et al., 2003 [31]	HF	72.5	63	NA	135	170	61	109	32	31	NA	NA
Gheorghiade et al., 2007 [32]	HF	NA	NA	NA	135	40454	7882	32572	473	1042	NA	NA
Gheorghiade et al., 2007 [33]	HF	56.2	NA	NA	134	430	103	327	31	52	NA	NA
Milo-Cotter et al, 2008 [34]	HF	74.9	51	NA	135	296	38	258	11	21	NA	NA
Tribouilloy et al., 2008 [35]	HF	74	53.8	25.8	NA	662	NA	NA	NA	NA	136.7±4.9	138.4±3.6
Rusinaru et al., 2009 [36]	HF	75.8	46.6	26.2	136	358	91	267	73	159	NA	NA
Barsheshet et al., 2010 [37]	HF	NA	55.3	51.7	136	2336	537	1799	54	74	NA	NA
DeWolfe et al., 2010 [38]	HF	54.7	62.9	34.1	135	364	48	316	8	31	NA	NA
Novack et al., 2010 [39]	HF	75.6	52.2	38.3	136	8246	1755	6491	NA	NA	136.4±5.3	137.6±4.5
Baldasseroni et al., 2011 [40]	HF	62	74.4	11.0	135	4670	463	4207	123	433	NA	NA
Balling et al., 2011 [41]	HF	68	73	NA	136	3645	602	2863	147	429	NA	NA
Shorr et al., 2011 [42]	HF	74.7	46.2	NA	135	115969	24562	91407	1372	2763	NA	NA
Arroyo et al., 1976 [43]	CIRRHOSIS	NA	NA	NA	130	55	21	34	9	6	NA	NA
Vila et al., 1999 [44]	CIRRHOSIS	47.3	35.2	NA	130	45	20	25	7	9	NA	NA
Borroni et al., 2000 [45]	CIRRHOSIS	56.9	70.5	NA	130	191	57	134	15	12	NA	NA
Porcel et al,. 2002 [46]	CIRRHOSIS	62.9	62.1	NA	130	74	54	20	37	5	123.8±5.6	129±7.7
Ruf et al., 2005 [47]	CIRRHOSIS	49	53	NA	130	194	34	160	NA	NA	130±6.0	136±5.0
Hackworth et al., 2009 [48]	CIRRHOSIS	51	78	NA	130	213	90	123	10	10	NA	NA
Radha Krishna et al., 2009 [49]	CIRRHOSIS	36.3	70.2	NA	NA	121	50	71	38	16	NA	NA
Terg et al., 2009 [50]	CIRRHOSIS	NA	NA	NA	130	81	27	54	12	7	NA	NA
Jenq et al., 2010 [51]	CIRRHOSIS	56	76.2	NA	135	126	67	59	49	33	NA	NA
Singhi et al., 1992 [52]	PNEUMOPATHY	3.14	NA	NA	135	727	371	356	24	17	NA	NA
Sharma et al., 1995 [53]	PNEUMOPATHY	35	51	NA	135	112	42	70	NA	NA	117.6±5.8	132.6±7.7
El-Ebiary et al., 1997 [54]	PNEUMOPATHY	59.1	75	17	136	84	9	75	6	19	NA	NA
Hussain et al., 2004 [55]	PNEUMOPATHY	47	42	15	135	110	78	32	NA	NA	127.8±7.4	130.6±7.5
Nair et al., 2007 [56]	PNEUMOPATHY	73.5	50	20	135	342	95	247	9	8	NA	NA
Song et al., 2008 [57]	PNEUMOPATHY	NA	NA	NA	NA	929	78	851	16	62	NA	NA
Zilberberg et al., 2008 [12]	PNEUMOPATHY	68.4	45.2	NA	135	7965	649	7316	35	293	NA	NA
Sunderam et al., 1983 [58]	AGED	NA	0	NA	130	683	108	575	53	104	NA	NA
Samadi et al., 1985 [59]	CHRONIC DIARRHEA	<3	NA	NA	130	1330	276	1054	28	38	NA	NA
Cusano et al., 1990 [60]	AIDS	36.6	89	NA	130	96	30	66	21	24	NA	NA
Vitting et., 1990 [61]	AIDS	39.8	98	NA	132	48	34	14	8	2	128±2	133±1
Erinoso et al., 1993 [62]	MALNUTRITION	<5	59.3	NA	130	120	85	35	40	6	NA	NA
Tang et al., 1993 [63]	AIDS	34.2	NA	NA	135	210	83	127	30	25	NA	NA
Terzian et al., 1994 [16]	AGED	>65	43.4	NA	130	4123	145	3978	23	316	NA	NA
Chuah et al., 1996 [64]	AMEBIASIS	NA	80	NA	135	60	23	37	15	1	NA	NA
Iseki et al., 1996 [65]	DIALYSIS	50.9	56.6	NA	NA	1491	NA	NA	NA	NA	134.9±7.6	136.8±5.8
Srivastava et al., 1998 [66]	FULMINANT HEPATITIS	5.3	68.3	NA	125	41	3	38	3	22	NA	NA
Berghmans et al., 2000 [67]	TUMOURS	NA	NA	NA	130	3306	106	3200	21	202	NA	NA
Manary et al., 2000 [68]	MALNUTRITION	2.7	45.3	NA	NA	75	NA	NA	NA	NA	131	132
Oguche et al., 2002 [69]	MALARIA	3	44	NA	NA	50	8	42	1	10	NA	NA
Agarwal et al., 2004 [70]	ACUTE RENAL FAILURE	<12	70	NA	NA	54	12	42	9	19	NA	NA
Lee et al., 2005 [71]	BONE MARROW TRANSPLANTATION	32	58.2	NA	134	311	185	126	123	43	NA	NA
Sherlock et al., 2006 [72]	SUBARACHNOID HEMORRHAGE	NA	NA	NA	136	316	179	137	19	26	NA	NA
Bonney et al., 2008 [73]	LIVER TRANSPLANTATION	52.8	49.1	NA	NA	54	17	37	5	5	NA	NA
Forfia et al., 2008 [74]	LUNG HYPERTENSION	55.3	17	NA	136	40	13	27	5	11	NA	NA
Olotu et al., 2008 [75]	HEMOLYTIC-UREMIC SYNDROME	NA	61	NA	120	31	8	23	7	10	NA	NA
Hanson et al., 2009 [76]	MALARIA	35	79.5	NA	135	168	98	70	31	36	NA	NA
Hsu et al., 2009 [77]	TUMOUR LISYS SYNDROME	55.2	66.7	NA	NA	12	NA	NA	NA	NA	132±6	142±3
Kapoor et al., 2010 [78]	PYELONEPHRITIS	57	15.4	NA	120	39	15	24	5	0	NA	NA
Dimopoulos et al., 2010 [79]	CONGENITAL HEART DISEASE	36.2	48.7	NA	136	1004	156	848	35	61	NA	NA
Salvador et al., 2010 [80]	NECROTIZING FASCIITIS	NA	NA	22	135	67	14 53	53	6	18	NA	NA
Scherz et al., 2010 [81]	PULMONARY EMBOLISM	67	40.2	NA	135	13728	2907	10821	441	866	NA	NA
Stelfox et al., 2010 [82]	HEART SURGERY	65.4	76.4	41.9	133	6727	785	5942	82	124	NA	NA
Hoorn et al., 2011 [83]	AGED	70.3	38.5	11	136	5208	399	4809	206	1567	NA	NA
Saifudheen et al., 2011 [84]	GUILLAIN BARRE SYNDROME	42	72	NA	135	50	24	26	4	0	NA	NA
Vaa et al., 2011 [85]	ALCOHOLIC HEPATITIS	51.1	85	NA	NA	26	NA	NA	NA	NA	132	136
Tierney et al., 1986 [86]	HOSPITALIZED SERIES	61.2	47	19	135	1514	757	757	165	60	NA	NA
Natkunam et al., 1991 [87]	HOSPITALIZED SERIES	NA	NA	NA	125	1217	202	1015	84	35	NA	NA
Singhi et al., 1994 [88]	HOSPITALIZED SERIES	NA	75	NA	135	264	71	193	6	7	NA	NA
Miller et al., 1995 [89]	HOSPITALIZED SERIES	60–103	91.6	NA	135	119	63	56	11	12	NA	NA
Gill et al., 2006 [3]	HOSPITALIZED SERIES	65	47.5	NA	125	204	104	100	28	9	NA	NA
Asadollahi et al., 2007 [90]	HOSPITALIZED SERES	NA	NA	NA	134	1599	356	1243	179	377	NA	NA
Stelfox et al., 2008 [17]	HOSPITALIZED SERIES SERIES	56.1	58.9	NA	133	5985	917	5068	255	799	NA	NA
Zilberberg et al, 2008 [91]	HOSPITALIZED SERIES	61.8	45.5	NA	135	198281	10899	187382	643	5621	NA	NA
Hampshire et al., 2009 [92]	HOSPITALIZED SERIES	NA	NA	NA	130	6410	285	6125	208	3468	NA	NA
Whelan et al., 2009 [93]	HOSPITALIZED SERIES	58.5	47.5	NA	134	14039	2795	11244	474	893	NA	NA
Whyte et al., 2009 [94]	HOSPITALIZED SERIES	68.8	39.8	NA	120	226	113	113	24	7	NA	NA
Funk et al., 2010 [95]	HOSPITALIZED SERIES	63.2	57.6	NA	135	140952	26782	114170	4369	11074	NA	NA
Wald et al., 2010 [11]	HOSPITALIZED SERIES	65.3	48.2	14.9	138	34761	13274	21487	451	430	NA	NA
Chawla et al., 2011 [18]	HOSPITALIZED SERIES	NA	NA	NA	135	209839	45693	164146	2787	3775	NA	NA

H: patients with hyponatremia; NH: patients without hyponatremia; DM: diabetes mellitus; NA: not available.

The mean±SD serum [Na^+^] in dead or alive individuals was specified in 3 of the aforementioned studies and in a further 8 studies enrolling patients with HF (n = 2), cirrhosis (n = 1), pulmonary infection (n = 2) or mixed disease (n = 3), respectively (Table 1).

Overall 850222 patients and 147948 hyponatremic subjects were included in the meta-analysis. Hyponatremia was defined according to varying cut-off definitions in the included studies (Table 1). The Begg-adjusted rank correlation test, calculated on the basis of overall mortality rate for hyponatremia, suggested no major publication bias (Kendall tau 0.02; p = 0.82).

**Figure 2:**
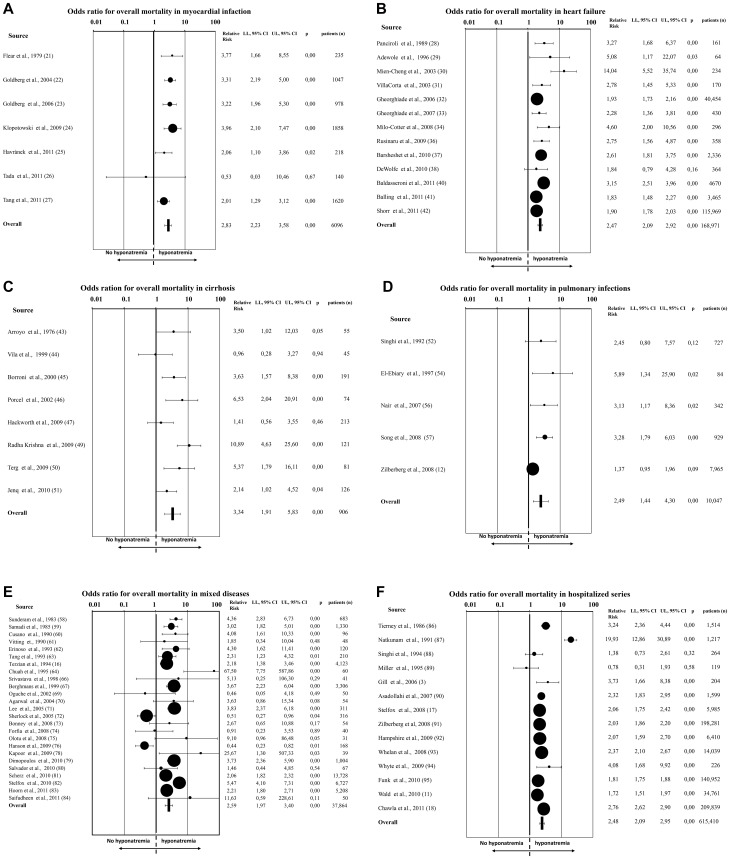
Odds ratio for overall mortality in patients with or without (no) hyponatremia according to the presence of myocardial infarction (A), heart failure (B), cirrhosis (C), pulmonary infection (D), mixed disease (E), or in hospitalized series of subjects (F).

**Figure 3:**
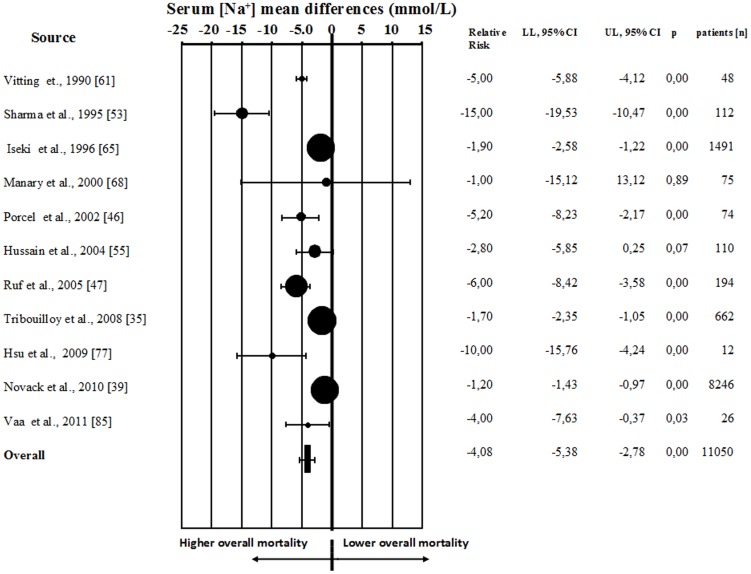
Weighted differences (with 95% CI) of mean serum [Na^+^] in dead and alive patients.

When all 81 studies were considered, hyponatremia was significantly associated with an increased risk of overall mortality (RR = 2.60[2.31–2.93]; p<0.0001). Similar results were obtained when patients with specific diseases or series of hospitalized patients were analyzed separately (Figure 2, panels A–E). Similar to what observed for mortality rate, the Begg-adjusted rank correlation test, calculated on the basis of mean serum [Na^+^] between subjects who eventually died when compared to survivors, suggested no major publication bias (Kendall tau −0.145; p = 0.553). The baseline mean difference of serum [Na^+^] was significantly lower in subjects who eventually died when compared to survivors (130.1±5.6 *vs* 134.9±5.1 mmol/L) at follow up (Figure 3). Similar results were observed when studies enrolling less than 100 subjects were excluded from the analysis (mean difference in serum [Na^+^] between survivors *vs* dead 3.04[1.81–4.27], p<0.0001). Sub-analysis for mean serum [Na^+^] in specific diseases was not performed due to insufficient data.

**Figure 4:**
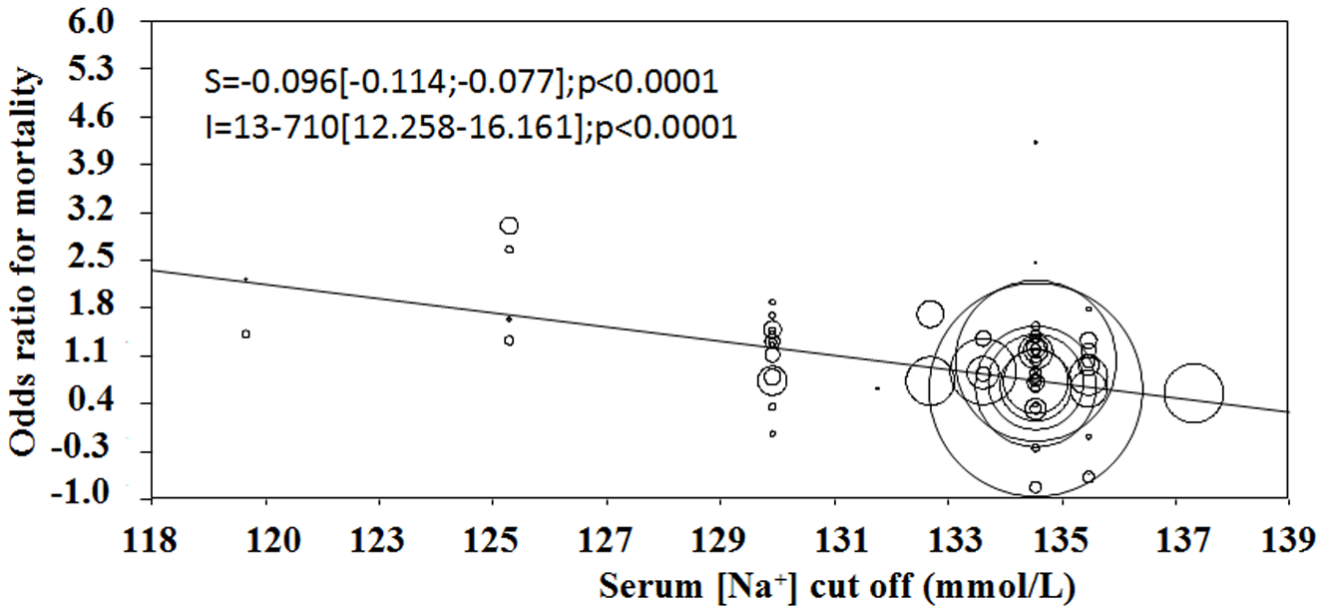
Relation between serum [Na^+^] cut-off definition and overall mortality risk.

A meta-regression analysis showed that the hyponatremia-related risk of overall mortality was inversely correlated with the serum [Na^+^] threshold considered for each report (Figure 4). Hence, the lower threshold considered, the higher the risk of mortality. The latter association was confirmed in a multiple regression model, adjusting for age, gender and diabetes mellitus (adj. r = −0.278; p<0.0001).

Sensitivity analyses performed considering only larger studies (including ≥1000 subjects), those reporting the prevalence of diabetes mellitus or those with severe hyponatremia ([Na^+^] ≤125 mmol/l), confirmed the association between hyponatremia and mortality (RR = 2.521[2.180–2.916]; p<0.0001 and 2.886[2.228–3.737], 10.036[5.155–19.540]; all p<0.0001, respectively).

## Discussion

Hyponatremia has been associated with increased in-hospital mortality [11], but no published comprehensive meta-analysis that analyzed the mortality rate in subjects with or without hyponatremia had been performed to date. Very recently, the Meta-Analysis Global Group in Chronic heart failure (MAGGIC) published a meta-analysis that included 14766 patients from 22 studies that recruited patients with HF and reported death from any cause [19]. Patients with hyponatremia (n = 1618) had an increased risk of death (21%), compared to patients with normal serum [Na^+^] (16%), and the risk of death appeared to increase linearly with serum [Na^+^] <140 mmol/L. Hyponatremia was an independent predictor of death either when the patients were considered as a whole, or when they were grouped based on the presence of a reduced (n = 1199) or a preserved (n = 419) ejection fraction. The MAGGIC meta-analysis was limited to patients with HF and considered studies published to the end of 2008.

Our meta-analysis included all of the English-language published studies up until October 1^st^ 2012 that compared the mortality rate in human subjects with or without hyponatremia of any degree. Eighty-one published studies were selected according to specified inclusion criteria for a total of 850222 patients, of whom 17.4% were hyponatremic. This percentage is in general agreement with epidemiological data about the prevalence of hyponatremia among hospitalized patients [2]. Of note, hyponatremia was associated with a significantly increased risk of overall mortality when all studies were considered (RR = 2.60 [2.31–2.93]). A detailed analysis of cause specific mortality was not possible, because this information was not available in several studies, as also was found in the MAGGIC meta-analysis. Nevertheless, we were able to conclude that the risk of mortality was independent of factors including age, gender, and diabetes mellitus as an associated morbidity. Similarly, hyponatremia was found to be associated with an increased risk of death when the patients were analyzed separately based on different disease types or when sensitivity analysis was restricted to larger studies or those reporting the prevalence of diabetes. In particular, we were able to confirm the data of the MAGGIC meta-analysis on hyponatremic patients with HF (RR = 2.47 [2.09–2.92]), analyzing a greater number of patients (168971, of whom 20.4% were hyponatremic). In the MAGGIC meta-analysis, only 11% of patients were hyponatremic, which is below the prevalence of hyponatremia generally reported for hospitalized patients (15–30%) [2]; the authors suggested that this might be due to the fact that all patients in the MAGGIC cohort were outpatients at the time of the baseline data. In contrast with the MAGGIC meta-analysis, patients with hyponatremia in our meta-analysis were neither older, nor more frequently affected by diabetes mellitus. Furthermore, we found an increased risk of mortality in hyponatremic patients with myocardial infarction (total number of patients 6096, of whom 18.3% with hyponatremia), cirrhosis (total number of patients 906, of whom 42.6% were hyponatremic), or pulmonary infections (total number of patients 10047, of whom 12% were hyponatremic). Some studies (n = 26) reported data regarding other mixed diseases or subpopulations (e.g., elderly people), which could not be grouped together. The most represented diseases among these patients (total number of patients 37864, of whom 15.1% were hyponatremic) were AIDS, malaria and malnutrition. Finally, some studies (n = 14, total number of patients 615410, of whom 16.7% were hyponatremic) were considered separately, because the effect of hyponatremia on mortality was investigated retrospectively and the diagnoses were not specified. The meta-analysis of these studies also revealed an increased risk of overall mortality.

The major finding of this meta-analysis is that across all groups of patients the relative risk of mortality in patients with hyponatremia *vs* patients without hyponatremia ranged between 2.47 and 3.34, thus indicating that this electrolyte disorder strongly predicts prognosis of all hospitalized patients. Another interesting result of our meta-analysis is that a moderate serum [Na^+^] reduction (i.e., 4.8 mmol/L) was associated with an increased risk of mortality, and a meta-regression analysis showed that the hyponatremia-related risk of overall mortality was inversely correlated with the serum [Na^+^]. Hence, the lower threshold considered, the higher the risk of mortality. This association was confirmed in a multiple regression model after adjusting for age, gender and diabetes mellitus. The linear increase of risk of death that we showed in our analysis is in agreement with the findings of the MAGGIC meta-analysis, which found a linear increase of mortality starting at serum [Na^+^] <140 mmol/L. Overall, our findings indicate that even a moderate reduction of serum [Na^+^] is associated with an increased risk of mortality in patients affected by multiple disease types across large numbers of hospitalized patients.

Although the present meta-analysis both confirms and extends the strong association between hyponatremia and adverse outcomes such as inpatient mortality, it cannot prove a causal relation between these variables. In fact, only diabetes mellitus could be used as a possible confounder in the present study. Perhaps the major outstanding question regarding hyponatremia is whether hyponatremia contributes directly to poor outcomes or is simply a marker for severity of underlying co-morbidities, or possibly for other factors that might influence the progression of underling co-morbidities [96]. Hence, it should be recognized that potential unmeasured confounders such as other chronic diseases, in addition to diabetes mellitus, may have caused residual confounding, but the measured factors that are correlated with such confounders would have mitigated the bias. Few studies to date have attempted to address the issue of a direct effect of hyponatremia on mortality or other adverse outcomes. One oft-cited potential exception is the Efficacy of Vasopressin Antagonism in Heart Failure Outcome Study with Tolvaptan (EVEREST) study of patients with congestive heart failure, which failed to show improvements in cardiovascular outcomes in patients with acute heart failure (AHF) treated with the vasopressin type 2 receptor (V2R) antagonist, tolvaptan, versus placebo [97]. However, that study was not powered to examine outcomes in the smaller subgroup of patients enrolled with both heart failure and hyponatremia. More recently, a significant strong positive relationship between an increase in serum sodium and decreased mortality was noted in 322 patients hospitalized for AHF and followed for 1–3 years [98]. In contrast, a multicenter analysis of 2888 patients hospitalized for AHF in Korea confirmed that hyponatremia on admission was associated with a worse prognosis compared with normonatremia, but this relation persisted regardless of whether the hyponatremia improved during the hospitalization [99]. However, this report was a retrospective anaylsis from a registry, not a prospective randomized trial, and the assessment of the change in serum sodium was made only once, prior to or at discharge from the hospital [100]. Thus, whether hyponatremia is merely a marker or also a mediator of adverse patient outcomes is still uncertain in heart failure, and has not been studied in other diseases. The current meta-analysis adds further urgency to the need to answer this question for multiple diseases, not only heart failure.

In conclusion, this study represents the first extensive and updated meta-analysis demonstrating that hyponatremia is significantly associated with an increased risk of overall mortality, and that it is a negative prognostic factor across multiple commonly observed clinical conditions, such as myocardial infarction, HF, cirrhosis and pulmonary infections. These findings might suggest the importance to correct this electrolyte disorder, even when mild, using the most appropriate strategies [101]–[103]. However, our study did not specifically address this issue and this hypothesis at present highlights the need for additional studies of clinical outcomes with effective therapies in all hyponatremic patients.

## Supporting Information

Checklist S1 **PRISMA Checklist.**
(DOC)
