# Supplementary material for: Moderate Hyponatremia Is Associated with Increased Risk of Mortality: Evidence from a Meta-Analysis
Source: PLoS One. 2013 Dec 18;8(12):e80451. doi: 10.1371/journal.pone.0080451 (PMC3867320; doi:10.1371/journal.pone.0080451)
Supplement: Checklist S1 — PRISMA Checklist. (DOC) [file pone.0080451.s001.doc]

| **Section/topic** | **#** | **Checklist item** | **Reported on page #** |
| --- | --- | --- | --- |
| **TITLE** | | |  |
| Title | 1 | MODERATE HYPONATREMIA IS ASSOCIATED WITH AN INCREASED RISK OF MORTALITY: A COMPREHENSIVE REVIEW AND META-ANALYSIS | Title page |
| **ABSTRACT** | | |  |
| Structured summary | 2 | Background: Hyponatremia is the most common electrolyte disorder in clinical practice, and evidence to date indicates that severe hyponatremia is associated with increased morbidity and mortality. The aim of our study was to perform a meta-analysis that included the published studies that compared mortality rates in subjects with or without hyponatremia of any degree.  Methods and findings: An extensive Medline, Embase and Cochrane search was performed to retrieve the studies published up to October 1st 2012, using the following words: “hyponatremia” and “mortality”. Eighty-one studies satisfied inclusion criteria encompassing a total of 850222 patients, of whom 17.4% were hyponatremic. The identification of relevant abstracts, the selection of studies and the subsequent data extraction were performed independently by two of the authors, and conflicts resolved by a third investigator. Across all 81 studies, hyponatremia was significantly associated with an increased risk of overall mortality (RR=2.60[2.31-2.93]). Hyponatremia was also associated with an increased risk of mortality in patients with myocardial infarction (RR=2.83[2.23-3.58]), heart failure (RR=2.47[2.09-2.92]), cirrhosis (RR=3.34[1.91-5.83]), pulmonary infections (RR=2.49[1.44-4.30]), mixed diseases (RR=2.59[1.97-3.40]), and in hospitalized patients (RR=2.48[2.09-2.95]). A mean difference of serum [Na+] of 4.8 mmol/L was found in subjects who died compared to survivors (130.1±5.6 vs 134.9±5.1 mmol/L). A meta-regression analysis showed that the hyponatremia-related risk of overall mortality was inversely correlated with serum [Na+]. This association was confirmed in a multiple regression model after adjusting for age, gender, and diabetes mellitus as an associated morbidity.  Conclusions: This meta-analysis shows for the first time that even a moderate serum [Na+] decrease is associated with an increased risk of mortality in commonly observed clinical conditions across large numbers of patients. | Abstract |
| **INTRODUCTION** | | |  |
| Rationale | 3 | Hyponatremia is the most common electrolyte disorder in clinical practice, and evidence to date indicates that severe hyponatremia is associated with increased morbidity and mortality. Whether hyponatremia is an independent risk factor for death or is simply associated with an underlying severe condition that is the cause of death remains to be elucidated. In addition, the role of milder forms of hyponatremia is conflicting. | Introduction |
| Objectives | 4 | To review and meta-analyse all the studies that compared the mortality rate in subjects with or without hyponatremia, in order to verify whether hyponatremia represents a risk factor for mortality, independently of other confounding factors. | Introduction |
| **METHODS** | | |  |
| Protocol and registration | 5 | We included case-control studies, prospective cohort studies, cross-sectional studies, comparisons of study populations with age standardization; | Methods |
| Eligibility criteria | 6 | The research was restricted to English-language articles and studies of human participants. | Methods |
| Information sources | 7 | PubMed from 1965 – October 1st 2012  EMBASE from 1974 – October 1st 2012  Cochrane from 1967 – October 1st 2012. | Methods |
| Search | 8 | An extensive Medline, Embase, and Cochrane search was performed including the following words: hyponatremia and mortality. | Methods |
| Study selection | 9 | We did not employ a search software. We hand-searched bibliographies of retrieved papers for additional references  Details of the literature search process are outlined in the flow chart (Figure 1). | Methods |
| Data collection process | 10 | Data extraction were performed independently by two of the authors (G.P., C.G.), and conflicts resolved by a third investigator (G.C). The credentials of all investigators are indicated in the author list. | Methods |
| Data items | 11 | Data extracted from each of the studies were relevant to the population characteristics, study design, exposure, outcome, and possible effect modifiers of the association. | Methods |
| Risk of bias in individual studies | 12 | Quality of the studies was assessed using the Cochrane criteria. | Methods |
| Summary measures | 13 | Data were reported as adds ratios (ORs) with 95% CIs for hyponatremia-related mortality. In addition, when available, the baseline mean difference of serum [Na+] in subjects who eventually died when compared to survivors at follow up were also reported. | Methods |
| Synthesis of results | 14 | Heterogeneity was assessed using the I2 statistics for overall mortality rate. Considering that heterogeneity could not be excluded (I2 =92.8%), relative risk of mortality between subjects with or without hyponatremia, was calculated using both a random and fixed effect model. For a more conservative approach, results of random effect models were presented. | Methods |

Page 1 of 2

| **Section/topic** | **#** | **Checklist item** | **Reported on page #** |
| --- | --- | --- | --- |
| Risk of bias across studies | 15 | The Begg-adjusted rank correlation test, calculated on the basis of overall mortality rate for hyponatremia was used to test the presence of possible bias across studies. | Methods |
| Additional analyses | 16 | A meta-regression analysis was performed to test the effect of hyponatremia cut-off definition selected in the different studies on overall mortality rate levels. In addition, a linear multivariate regression analysis model, weighting each study for the number of subjects enrolled, was performed to verify the independent effect of hyponatremia on mortality after the adjustment for age, sex and diabetes mellitus. In addition, sensitivity analyses were performed considering only larger studies (including ≥1000 subjects) or those reporting the prevalence of diabetes mellitus. | Methods |
| **RESULTS** | | |  |
| Study selection | 17 | Out of 718 retrieved articles, 637 articles were excluded for different reasons summarized in Figure 1. In particular, we excluded studies that not reported mortality rate according to hyponatremia thresholds and or those which did not specified mean [Na+] in dead or alive subjects. | Results |
| Study characteristics | 18 | Quality of the studies was assessed using the Cochrane criteria. The specific characteristics of the studies included were summarized in Table 1. | Results |
| Risk of bias within studies | 19 | For a more conservative approach, results of random effect models were presented. | Results |
| Results of individual studies | 20 | We included 2 forest plot of all studies, 1 forest plot to examine effect [Na+] in predicting mortality (meta-regression analysis). | Results |
| Synthesis of results | 21 | When all 81 studies were considered, hyponatremia was significantly associated with an increased risk of overall mortality (RR=2.60[2.31-2.93]; p<0.0001). Similar results were obtained when patients with specific diseases or series of hospitalized patients were analyzed separately (Figure 2, panels A-E). The baseline mean difference of serum [Na+] was significantly lower in subjects who eventually died when compared to survivors (130.1±5.6 vs 134.9±5.1 mmol/L) at follow up (Figure 3). | Results |
| Risk of bias across studies | 22 | The Begg-adjusted rank correlation test, calculated on the basis of overall mortality rate for hyponatremia, suggested no major publication bias (Kendall tau 0.02; p=0.82). Similar to what observed for mortality rate, the Begg-adjusted rank correlation test, calculated on the basis of mean serum [Na+] between subjects who eventually died when compared to survivors, suggested no major publication bias (Kendall tau -0.145; p=0.553). | Results |
| Additional analysis | 23 | Sensitivity analyses confirmed the association between hyponatremia and overall mortality. A meta-regression analysis showed that the hyponatremia-related risk of overall mortality was inversely correlated with the cut-off definition of hyponatremia considered for each report (Figure 4). Hence, the lower threshold considered the higher the risk for mortality. The latter association was confirmed in a multiple regression model, adjusting for age, sex and associated morbidities including diabetes mellitus | Results |
| **DISCUSSION** | | |  |
| Summary of evidence | 24 | Across all studies, hyponatremia was significantly associated with an increased risk of overall mortality (RR=2.60[2.31-2.93]). These results were confirmed when specific diseases including myocardial infarction, heart failure, cirrhosis, pulmonary infections, mixed diseases or hospitalized patients were considered. In addition, a mean difference of serum [Na+] of 4.8 mmol/L was found in subjects who died compared to survivors (130.1±5.6 vs 134.9±5.1 mmol/L). Meta-regression analysis showed that the hyponatremia-related risk of overall mortality was inversely correlated with serum [Na+]. | Discussion |
| Limitations | 25 | We discussed the results of the sensitivity analyses. We recognized that potential unmeasured confounders such as other chronic diseases may have caused residual confounding. | Discussion |
| Conclusions | 26 | Our results indicates that even moderate hyponatremia is associated with a significant increased risk of overall morality in several clinical conditions. We recommend future studies on the effect of hypoantremia severity on mortality risk. | Discussion |
| **FUNDING** | | |  |
| Funding | 27 | No separate funding was necessary for the undertaking of this systematic review and meta-analysis |  |

*From:*  Moher D, Liberati A, Tetzlaff J, Altman DG, The PRISMA Group (2009). Preferred Reporting Items for Systematic Reviews and Meta-Analyses: The PRISMA Statement. PLoS Med 6(6): e1000097. doi:10.1371/journal.pmed1000097

For more information, visit: **www.prisma-statement.org**.

Page 2 of 2
